# Genomic Characterisation of Vinegar Hill Virus, An Australian Nairovirus Isolated in 1983 from *Argas Robertsi* Ticks Collected from Cattle Egrets

**DOI:** 10.3390/v9120373

**Published:** 2017-12-05

**Authors:** Penelope J. Gauci, Jane McAllister, Ian R. Mitchell, Daisy Cybinski, Toby St George, Aneta J. Gubala

**Affiliations:** 1 Land Division, Defence Science & Technology Group, Fishermans Bend, Victoria 3207, Australia; jane.mcallister@dst.defence.gov.au (J.M.); Ian.Mitchell@dst.defence.gov.au (I.R.M.); Ania.Gubala@dst.defence.gov.au (A.J.G.); 2 Formerly: Long Pocket Laboratories, Commonwealth Scientific and Industrial Research Organisation, Indooroopilly, Queensland 4068, Australia; dcybinski@hotmail.com (D.C.); tobystgeorge@bigpond.com (T.S.G.)

**Keywords:** *Bunyavirales*, *Nairoviridae*, *Orthonairovirus*, virus, ticks, genome, characterisation, Australia

## Abstract

This report describes the near complete genomic sequence and subsequent analysis of Vinegar Hill virus (VINHV; tentative member of the genus *Orthonairovirus*, family *Nairoviridae*, order *Bunyavirales*). VINHV is the second nairovirus reported to be isolated on mainland Australia and the first to be sequenced and analysed. Our genetic analysis shows that VINHV belongs to the Dera Ghazi Khan genogroup, a group of viruses previously isolated in other parts of the world including Asia, South Africa, and the USA. We discuss possible routes of entry for nairoviruses into Australia and the need to understand the virome of Australian ticks in the context of new and emerging disease.

## 1. Introduction

The genus *Orthonairovirus* (family *Nairoviridae*, order *Bunyavirales*) comprises 12 species to which more than 60 predominantly tick-borne viruses have been assigned, including several associated with severe human and livestock disease such as Crimean-Congo haemorrhagic fever (CCHF) and Nairobi sheep disease (NSD) viruses, respectively [1,2,3]. The nairovirus genome consists of three negative-sense, single-stranded RNA (-ssRNA) segments; small (S), medium (M), and large (L), that encode the nucleoprotein (N protein), glycoprotein precursor (GPC), and the RNA-dependant RNA polymerase (L protein), respectively. Until recently, there was little genetic data available for viruses of this genus, and the available information was mostly restricted to viruses of the CCHF and NSD serogroups. However, with the recent publication of several full-length nairoviruses genomes, our knowledge is increasing and changing the way we relate members within this important genus [3,4,5]. Consequently, Walker et al. [5] proposed the assignment of nairoviruses into nine distinct genogroups. For the most part, the genogroups represent the corresponding established serogroups, with the exception of the NSD and CCHF serogroups, which are combined into a single genogroup, NSD.

Nairoviruses have rarely been isolated in Australia or its territories. The isolation of three Australian nairoviruses has previously been documented; two Sakhalin genogroup viruses (Taggert virus, TAGV; and Finch Creek virus, FCV) isolated from *Ixodes uriae* ticks on Macquarie Island 1500 km south-southeast of Tasmania [6,7], and a tentative Dera Ghazi Khan (DGK) genogroup virus (isolate NT15470) isolated from *Argas robertsi* ticks in the Northern Territory [8]. Whilst TAGV is the only Australian nairovirus that has been fully sequenced [5], a partial sequence of FCV is also available in GenBank. An additional nairovirus, isolate CSIRO1499, which we describe in this manuscript and propose the name Vinegar Hill virus (VINHV), was isolated on the Australian mainland in 1983 [9]. VINHV was isolated from a pool of five female *A. robertsi* ticks collected from a rookery of the cattle egret (*Bulbulcus ibis*) at Gatton, Queensland in December 1981. The ticks were collected during an investigation of a heavy mortality in nestling chicks in the rookery. Following collection, the ticks were held alive until their blood meal was fully digested, and were subsequently stored frozen for future virus isolation. In contrast to the high isolation rate usually experienced with bird tick viruses, VINHV was isolated from only one of several hundred pools of ticks processed. Although results are unavailable, historical information suggests that serological testing found VINHV to be unrelated to any other arboviruses in Australia [9]. It is not evident from the literature if this testing included isolate NT15470, which had been demonstrated to be a strain of, or closely related to, Kao Shuan virus of the DGK genogroup [8]. VINHV was subsequently sent for further testing to the former World Reference Centre for Arboviruses at Yale (now the World Reference Centre for Emerging Viruses and Arboviruses at Galveston, TX, USA). Antibodies to VINHV have been detected in 13 of 401 (3.2%) tested seabird sera and 1 of 101 (1%) tested human sera. VINHV has been shown to produce an antibody response in experimentally infected cattle egrets and is capable of causing death in birds; however, no data on the pathogenicity of this virus for mammalian hosts is available [10].

The threat of new and emerging disease, together with the ease and availability of next generation sequencing technologies, has seen an increase in the characterisation of new and historic viruses. Here we report the near complete genome sequence of VINHV and subsequent predictive genetic and amino acid analyses, and demonstrate that this virus is a tentative member of the family *Nairoviridae*, genus *Orthonairovirus*. This is the first report describing the sequence of a nairovirus from the Australian mainland.

## 2. Materials and Methods

### 2.1. Virus Culture and Genomic Sequencing

Vinegar Hill virus (VINHV; isolate CSIRO1499) was obtained from the Berrimah Veterinary Laboratories, Darwin, NT, Australia. Viruses were propagated in baby hamster kidney BHK-BSR cells (a derivative of the BHK-21 cell line) grown in supplemented Basal Medium Eagle (Gibco, Grand Island, NY, USA) at 37 °C, following which they were harvested, and total RNA was extracted and converted to double stranded cDNA as previously described [11]. The cDNA material was prepared for high-throughput sequencing using the TruSeq CHIP-seq (Illumina, San Diego, CA, USA) protocols and standard multiplex adaptors. A paired-end, 250-base-read protocol was used for sequencing on an Illumina MiSeq instrument at The Ramaciotti Centre for Genomics, University of New South Wales, Sydney, Australia. Primary assembly of raw data and generation of consensus sequences were performed as described previously [11].

### 2.2. Bioinformatic Analysis

Alignments and predictive open reading frame (ORF) analysis was performed using Clone Manager (SciEd, Denver, CO, USA). Analysis of deduced proteins and targeting predictions were generated using the Centre for Biological Sequence Analysis portal tools including SignalP 4.1, NetOGlyc 4.0, NetNGlyc 1.0, ProP 1.0, and TMHMM (http://www.cbs.dtu.dk/services/). Pairwise identities were determined using *p*-distance analysis in MEGA7 [12].

### 2.3. Phylogenetic Analysis

Phylogenetic trees were constructed using 38 complete L protein, GPC, and N protein sequences, of nairoviruses accessed from GenBank (listed in Table S1) and the cognate VINHV protein sequences. Sequences were aligned using the MUSCLE 3.6 algorithm [13]. Bayesian analyses of amino acid (aa) sequence alignments were performed with BEAST software [14], using a Whelan and Goldman (WAG) model of aa substitution with gamma + invariant site heterogeneity. A lognormal relaxed clock model was also used, with a tree prior set to coalescent:exponential growth. The model was run with a Markov-chain Monte Carlo (MCMC) chain length of 10,000,000 with the output logged every 1000 steps, producing 10,000 trees. The maximum clade credibility tree was chosen using Tree Annotator (1000 tree burn-in) and trees were created using FigTree v1.4 (http://tree.bio.ed.ac.uk/software/figtree/). Maximum likelihood (ML) trees were constructed using MEGA5 [15], employing the WAG model of aa substitution with a gamma distribution of rate variation and 1000 bootstrap replications.

## 3. Results and Discussion

### 3.1. VINHV Genome and Terminal Sequences

The complete S and L genomic segments of VINHV and near complete M genomic segment were sequenced using high throughput sequencing (GenBank Accession numbers MF176883, MF176881, and MF176882, respectively). The lengths of the S, M, and L segments are 1729 nucleotides (nt), 4473 nt (lacking the 3′-terminal region, genome sense), and 12,133 nt, respectively. The organisation of the VINHV genome is consistent with those found in other orthonairoviruses, each containing a single ORF encoding the N protein, GPC, and L protein (Table 1). BlastX analysis of the GenBank databases indicates that VINHV is most similar to Dera Ghazi Khan virus (DGKV) sharing 78%, 69% and 71% amino acid identity with the translated protein products from the L, M, and S segments, respectively.

A common feature of bunyaviruses is the conservation of genus-specific genome termini. In nairoviruses, the consensus terminal nt sequences are 3′ AGAGUUUCU- and 5′ UCUCAAAGA-. The genome termini of VINHV S and L segments are consistent with this, with the exception of a single nucleotide change (U→A) at position 9 of the 3′ terminus of the S segment. The terminal sequences of Dera Ghazi Khan genogroup viruses have been observed to differ at position 9 of both terminal ends for each segment, with the exception of DGKV (Figure S1) [3]. DGKV has a deviation from the consensus at position 9 of the 3′ termini only of the M and S segments, similar to the observation in VINHV, further supporting a close relationship between VINHV and DGKV. Attempts to obtain the 3′ terminal non-coding sequence of the VINHV M segment were unsuccessful, despite several attempts. As the coding sequence for the GPC was complete, allowing comparative analyses with other GPCs to be performed, further attempts to obtain the non-coding portion at the 3′ terminus were abandoned. It is anticipated, however, that given the overall similarity of VINHV to DGKV, a similar sequence would be present at the 3′ end, though this would need to be confirmed.

### 3.2. L Protein

**Figure 1:**
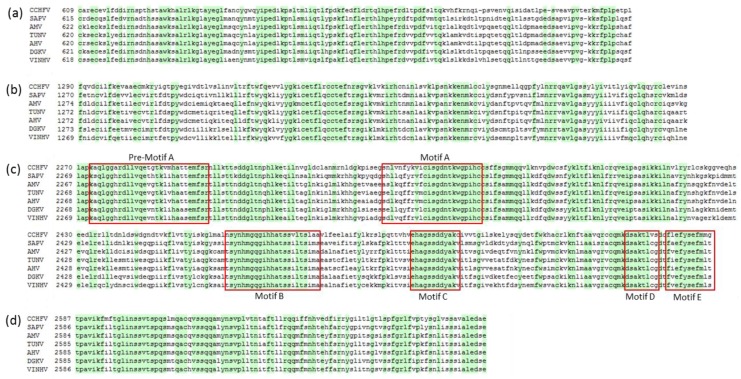
Conserved motifs on Vinegar Hill virus (VINHV) L protein. Amino acid sequence alignments of RNA-dependent RNA polymerase modules of VINHV and other Dera Ghazi Khan group viruses, with Crimean Congo haemorrhagic virus included as a reference. (**a**) Region I and (**b**) Region II are highly conserved in bunya- and arenaviruses; (**c**) Region III, including pre-motif A and motifs A–E, is highly conserved in negative sense RNA viruses, and (**d**) Region IV is highly conserved in bunyaviruses and other segmented negative sense RNA viruses. Conserved amino acid residues amongst the DGK group of viruses and CCHFV are shaded green.

The single ORF on the L segment of VINHV encodes a 3948-aa viral L polymerase protein. The L polymerases of -ssRNA viruses contain four conserved regions reflective of the universal functions of this protein [16,17,18]. Region I has a presumed cap-snatching endonuclease activity [19], whilst the function of region II is unknown. Region III, also called the polymerase module, contains six conserved motifs (pre-motif A and motifs A–E) and is predicted to be involved in catalytic functions of the polymerase, and in template and/or primer positioning [17]. Region IV is suggested to have a role in capped primer-cleavage and 5′ viral RNA binding [16]. All of these regions and motifs are highly conserved in the putative VINHV L protein (Figure 1a–d). Although zinc finger and leucine zipper sequence motifs have previously been identified in the CCHFV L protein [20], these are not always apparent in all nairoviruses [3]. Likewise, they are not apparent in the VINHV L protein.

**Figure 2:**
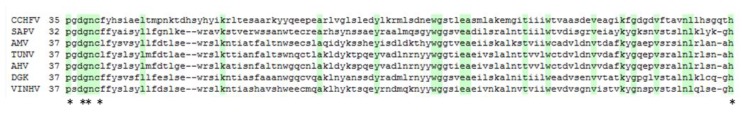
Ovarian tumour (OTU)-like domain on VINHV L protein. Amino acid sequence alignment of a fragment of the L protein of VINHV and other DGK group viruses depicting a predicted (OTU)-like protease domain close to the N-terminal region. Amino acids that constitute the domain are marked with * below the sequence. Amino acids conserved between the DGK group viruses and CCHFV are shaded green.

In addition to these regions, an ovarian tumour (OTU)-like domain (pfam02338) has been identified in proximity to the N termini of the L protein of all nairoviruses with the possible exception of the “nairo-like” viruses including South Bay virus, which has a divergent sequence that shows some homology to the OTU-like domain [3,5,20,21]. Similarly, an OTU-like domain is predicted in the VINHV L protein (Figure 2). The observed functionality differences between the OTU domains of virulent CCHFV and less virulent DUGV lead some to speculate that this domain may be a virulence factor [3,22].

### 3.3. GPC

**Figure 3:**
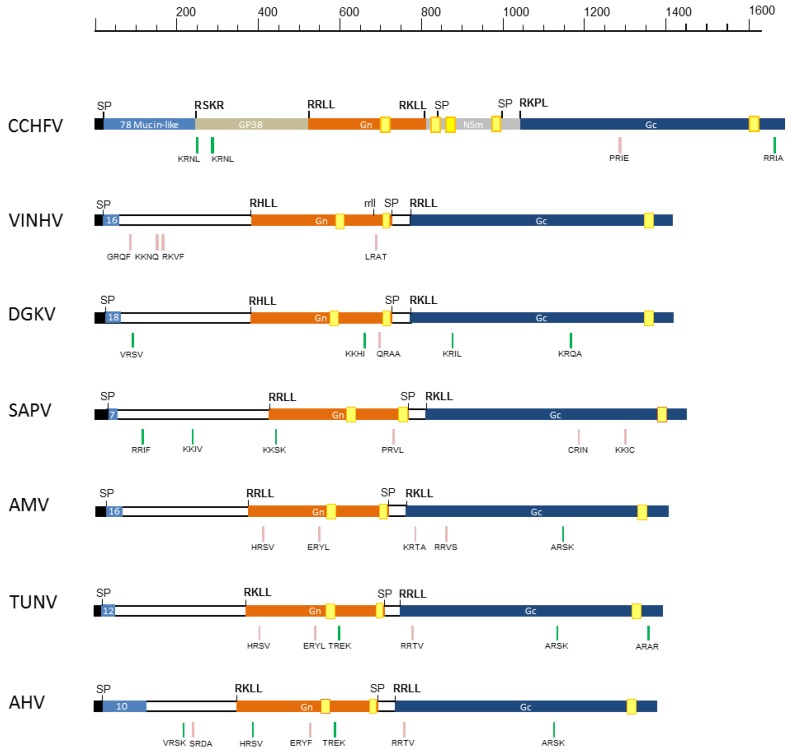
Illustration of the glycoprotein precursors (GPC) of VINHV and other DGK group nairoviruses. The CCHFV GPC is included as a reference for comparison. Regions corresponding to the mucin-like domain (light blue), GP38 (tan), Gn (orange), NSm (grey), and Gc (dark blue) are depicted. The numbers of predicted *O*-linked glycosylation sites in the mucin-like domain are shown. Signal peptidase cleavage sites (SP), potential furin-like (for CCHFV only) and subtilisin/kexin-isozyme-1 (SKI-I) cleavage sites that might be utilised to generate the individual proteins, were predicted using SignalP 4.1 and comparative studies and are shown above each schematic. A potential alternate cleavage site (rrll) identified in the VINHV protein is depicted in lower case. Transmembrane domains are indicated by yellow boxes. Additional cleavage sites predicted by ProP1.0 are indicated below the schematic with a green stripe (scores > 0.5, higher confidence) or a pink stripe (scores 0.3–0.49, lower confidence). DGKV: Dera Ghazi Khan virus; SAPV: Sapphire II virus; AMV: Abu Mina virus; TUNV: Tunis virus; AHV: Abu Hammad virus.

The VINHV M segment contains a single ORF that putatively encodes a 1414-aa polyprotein, which, like other bunyavirus M segment polyproteins, is predicted to be co- and post-translationally processed into mature viral glycoproteins [23]. The VINHV polyprotein shares similar sequence organisation to other nairoviruses and contains various conserved post-translational modification sites and structural features (Figure 3). The study of CCHFV provides much of our understanding of nairovirus GPC structure and processing [24,25,26,27,28]. The analysis of CCHFV shows that the GPC has an N-terminal mucin-like domain containing a large number of predicted *O*-glycosylation sites, followed by a protein of unknown function (GP38), an envelope glycoprotein (Gn), a non-structural protein (NSm), and a second envelope glycoprotein (Gc).

Similar to other nairoviruses, the VINHV GPC is predicted to contain an N-terminal signal peptide (at VLA_30_-NT) followed by a highly *O*-glycoslylated (16 sites) mucin-like domain, but one which is considerably shorter with less predicted *O*-glycosylation sites than in CCHFV. Although the function of the mucin-like domain of CCHFV GPC remains undetermined, a similar mucin-like domain in the Ebola virus glycoprotein GP1 is known to play a major role in pathogenesis [29]. The M segment is the most variable of the three segments, and this is particularly notable in the hypervariable N-terminal region that precedes the Gn protein. The characteristics of this region are generally genogroup-specific in relation to variation in the number of *O*-glycosylation sites and the length of the predicted mucin-like domain [5]. Viruses of the DGK genogroup are generally known to have one of the smallest mucin-like domains amongst the nairoviruses ranging between 56 to 124 aa in length, containing between seven and 22 *O*-glycosylation sites. The CCHFV mucin-like domain is cleaved by a furin or furin-like protease (site RSKR), generating a 247-aa protein [30]. There does not appear to be an equivalent furin-like protease cleavage site in any of the analysed DGK group GPCs, including in VINHV; however, there are a number of possible alternate protease cleavage sites in the vicinity of the domain (Figure 3). The VINHV M segment does not appear to encode an NSm protein, which is consistent with all other nairoviruses except for the NSD genogroup viruses, which do encode this protein [5].

The Gn and Gc glycoproteins of nairoviruses are relatively well conserved in size and structural characteristics [5]. Similar to predictions in other nairoviruses, the VINHV Gn and Gc proteins are predicted to be cleaved by the subtilisin/kexin-isozyme-1 (SKI-1) protease at sites RHLL_383_↓ and RRLL_775_↓, respectively. The VINHV Gn and Gc proteins are of similar size to those of other DGK group viruses, and they contain numerous conserved cysteine residues which have a functional role in protein folding, transmembrane domains, and zinc finger domains (Figure 3; Figures S2 and S3).

VINHV Gn contains three predicted glycosylation sites (Figure S2). The location of the first glycosylation site (NGTK_432_) is universally conserved amongst all nairoviruses. The second glycosylation site (NGSG_498_) appears to be conserved with DGKV and Sapphire II virus (SAPV), whilst the third site (NHTS_509_) appears to be unique to VINHV. Similarly, there are three predicted glycosylation sites in the VINHV Gc protein (Figure S3). The first (NNSV_795_) is conserved amongst all the analysed DGK viruses with the exception of SAPV, and the second (NGSI_1151_) is conserved in all the analysed DGK group viruses. The third site (NCTG_1309_) is generally conserved with most of the viruses in the orthonairovirus genus [5].

### 3.4. N Protein

The N protein of -ssRNA viruses binds to genomic RNA to form ribonucleoprotein complexes that associate with the polymerase for viral RNA synthesis (transcription and replication) and form the structural core of the virion [31]. The length of the VINHV N protein is 499 aa, which is similar to those of other nairoviruses. Crystal structure studies of the N protein of CCHFV demonstrated two major domains, a globular head and an extended stalk, with RNA/DNA binding-associated sites predominantly found on the head domain [31,32,33]. In comparative sequence analysis, the VINHV N protein exhibits conservation of these binding sites, either fully (K132, R134, K222, Q300, K343, R384, H453, and Q457), or with a conservative change (H197N, Y374H, E387D, and K411R) [31,32] (Figure S4). The caspase-3 cleavage site motif previously identified in some nairoviruses (CCHFV, Hazara virus (HAZV) and Thiafora genogroup viruses) is not apparent in the VINHV N protein [5].

Pairwise alignments show that the VINHV N protein shares 54.4 to 72% identity with the N proteins of other viruses within the DGK genogroup (Table 2) and 31.7 to 42.1% identity with the N proteins of representative viruses from the other genogroups (Table S2). Walker et al. [5] suggested a sequence identity cut-off of 52% to place viruses into genogroups. Using this criterion, the placement of VINHV into the Dera Ghazi Khan genogroup is well supported.

### 3.5. Phylogenetic Analysis

Until recently, only nairoviruses associated with hard ticks had been fully sequenced. This posed challenges for the phylogenetic analysis of nairoviruses associated with soft ticks, which were clearly different. Whilst partial L protein data for some soft tick nairoviruses has existed for some time [34], the recent work of Walker et al. [4,5] and Kuhn et al. [3] has produced sequence data enabling the comprehensive genomic analysis of numerous soft-tick viruses from this genus. It is evident from these studies that the phylogenetic relationship of nairoviruses broadly reflects vector preferences, genome organisation, and serological relationships.

**Figure 4:**
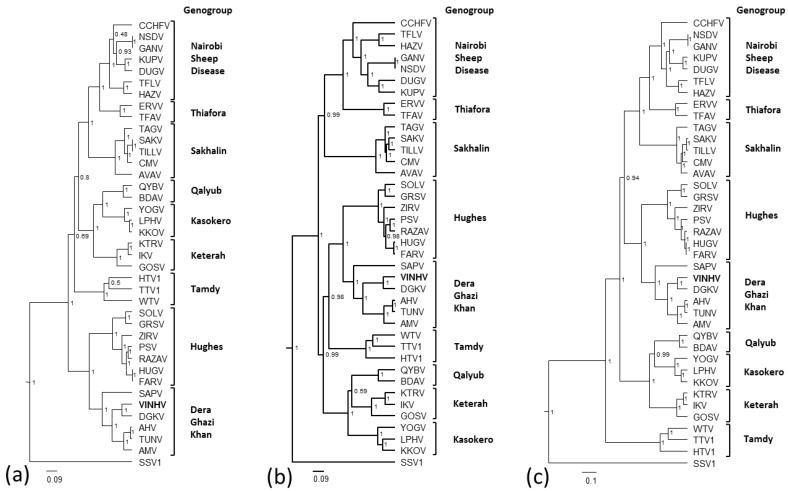
Phylogenetic analyses of the (**a**) nucleoprotein; (**b**) glycoprotein precursor, and (**c**) L protein of VINHV and other representative nairoviruses. Relationships were inferred by Bayesian analysis of the protein sequence alignments. A Whelan and Goldman (WAG) model of amino acid substitution with gamma + invariant site heterogeneity was used. Numbers represent Bayesian posterior probabilities. SSV1 was included as an outgroup. Trees are drawn to scale measured in substitutions per site as indicated by the scale bar.

Bayesian phylogenetic analyses were performed using the N protein, GPC, and L protein of VINHV and other representative nairoviruses (Figure 4a–c, respectively.) Maximum likelihood analyses were also performed for comparison and these produced trees with similar topologies (data not shown). The phylogenetic analyses demonstrate strong support for the formation of nine distinct clades representing the nine proposed genogroups [5]. Lower support present at some of the deeper nodes is most likely reflective of the divergence of some viruses, and will only be strengthened by the sequencing of additional viruses in this genus.

The inclusion of VINHV within the DGK genogroup is strongly supported and the relationships inferred within the group are uniform with all three segments. Viruses of the DGK genogroup are widespread throughout the world (Pakistan, Taiwan, Thailand, South Africa, USA, and Australia) and, in most instances, have been isolated from ticks feeding on birds. Thus, it is feasible that the distribution of this group of nairoviruses, including the introduction of VINHV and other nairoviruses to the Australian mainland, may be via avian migration. The cattle egret, which VINHV is associated with, has populated Australia only since the late 1940s.

Phylogenetic analyses also demonstrate that VINHV and DGKV share a common ancestor. DGKV was isolated from ticks feeding on camels in Pakistan in 1966. This may indicate another entry route for VINHV, or its ancestor, into Australia via the 10–20,000 camels that were brought into the country from India and Pakistan in the period of 1860–1907.

### 3.6. Ticks and Emerging Viruses in Australia

Though tick-borne diseases do not contribute greatly to the overall communicable disease burden in Australia, an increase in incidence may be seen in the future with climatic, population, and lifestyle changes [35]. Also, it is possible that a proportion of unknown or undiagnosed illnesses could be attributed to tick vectors. It is essential that we gain an understanding of the biome of the native ticks, particularly those that are known to bite humans. The *A. robertsi* tick, from which VINHV was isolated, is one of five soft tick species in Australia that possibly feed on humans and domestic animals [36]. However, the most important tick in Australia, from both a medical and veterinary perspective, is *Ixodes holocyclus*, a hard tick species. Hence, much of the tick research in Australia is focused on hard ticks and associated diseases, particularly of bacterial origin. *I. holocyclus* is the vector for *Rickettsia australis* and *R. honei*, the aetiological agents of the only two recognised tick-borne diseases in Australia—Queensland tick typhus and Flinders Island spotted fever, respectively [35,36]. It is speculated that this species of tick may also have a role in Hendra virus transmission [37]. Furthermore, amid debate regarding the presence of tick-borne Lyme disease in Australia, a *Borrelia* sp. related to the Lyme disease agent has been isolated from this species of tick [38]. However, despite patients presenting with Lyme-like disease, no aetiological agent has been linked to disease locally, and therefore the presence of Lyme disease in Australia is not confirmed.

Advances in sequencing technology have allowed us to investigate the biome of arthropods that are known vectors of disease. Although an ongoing study into tick-borne diseases in Australia has developed strategies to successfully identify low abundant bacteria in hard ticks [38], it is not evident whether this study will expand to include the identification of viral agents. Analysis of the viromes of three American ticks revealed a diverse array of viruses, including several novel viruses with genetic similarities to pathogens of humans and livestock [21]. Likewise, a similar metagenomics analysis of Australian mosquitoes detected the presence of viruses from families *Flaviridae*, *Rhabdoviridae*, *Reoviridae*, *Togaviridae*, and *Bunyaviridae* [39]. It is evident that there is great potential for novel and emerging viruses circulating in Australian arthropods.

Although some viruses have previously been isolated from Australian ticks [40,41], none as yet have been associated with human disease. However, it is important to note that antibodies to VINHV have been found in human sera and, as such, the potential threat to human health demands further investigation. With the ongoing sequencing of historic Australian virus isolates, our understanding of viruses circulating within Australia will also increase [4,5,11,41,42,43,44]. In other parts of the world, tick-borne infectious diseases are on the rise and becoming a serious world health problem affecting both human and animal health. For example, there has been a marked increase in the range and incidence of CCHF since 2000, and tick-borne encephalitis is a growing concern in Europe and Asia [45]. Similarly, the incursion of African swine fever into the Caucasus, and potentially from there into Europe, is of deep concern and requires preventative strategies to avoid the spread of this disease [46].

Bird migration plays an important part in the spread of tick-borne disease. For example, the massive expansion of the cattle egret range began after cattle became established in newly created, and expanding, cattle pastures in continents additional to Africa and Asia. They flew to the Americas in 1933, Australia 1948, and Europe in 1958, and have subsequently spread widely from there. Cattle were introduced to each of these new territories following European exploration and removal of forests. It is presumed that the associated ticks and tick-borne viruses have spread more slowly, as tick- and virus-free colonies exist within flight ranges of an infected colony. Thus, the pasture habitats are created for cattle, and the migrant egrets, ticks, and viruses follow, in that order. Investigations into the virome of Australian ticks will provide valuable information on the potential for the emergence of new viruses associated with this vector within the Australian landscape. Adequate biosurveillance in this area should be prioritised to mitigate any potential future emerging diseases.

## Figures and Tables

**Table 1:** Genome size comparisons of Vinegar Hill Virus (VINHV) and other Dera Ghazi Khan (DGK) group viruses (anti-genome sense).

Segment	Region	Length (nt/aa) for Indicated Viruses						
		VINHV	DGKV	AMV	AHV	TUNV	SAPV	^a^ CCHFV
L	5′UTR	53	57	41	35	35	96	76
	L ORF	11,847/3948	11,847/3948	11,925/3974	11,868/3955	11,868/3955	11,871/3956	11,837/3945
	3′UTR	233	106	249	214	400	218	194
	**segment total**	**12,133**	**12,010**	**12,215**	**12,117**	**12,303**	**12,185**	**12,108**
M	5′UTR	41 ^b^	42	14	30	30	14	92
	GPC ORF	4245/1414	4239/1412	4203/1400	4200/1399	4167/1388	4341/1446	5055/1684
	3′UTR	187	159	224	472	609	434	219
	**segment total**	**4473**	**4440**	**4441**	**4702**	**4806**	**4789**	**5366**
S	5′UTR	58	50	51	54	56	54	55
	NP ORF	1500/499	1500/499	1497/498	1497/498	1497/498	1476/491	1448/482
	3′UTR	171	221	212	226	315	137	168
	**segment total**	**1729**	**1771**	**1760**	**1777**	**1868**	**1668**	**1672**

^a^ CCHFV is included as a reference nairovirus. ^b^ Incomplete. VINHV: Vinegar Hill virus; DGK: Dera Ghazi Khan; UTR: Untranslated region; ORF: open reading frame; L: RNA dependent RNA polymerase; GPC: Glycoprotein precursor; NP: Nucleoprotein; DGKV: Dera Ghazi Khan virus; AMV: Abu Mina virus; AHV: Abu Hammad virus; TUNV: Tunis virus; SAPV: Sapphire II virus; CCHFV: Crimean Congo haemorrhagic virus.

**Table 2:** Amino acid sequence identities (%) of the nucleoproteins of Dera Ghazi Khan genogroup viruses. Sequence identities were determined by *p*-distance estimation using MEGA7.

Virus	VINHV	DGKV	AHV	TUNV	AMV
VINHV					
DGKV	72.0				
AHV	62.1	59.3			
TUNV	62.1	59.9	95.4		
AMV	60.6	63.4	76.0	75.8	
SAPV	54.4	52.9	52.4	52.4	52.4

## Supplementary Materials

The following are available online at www.mdpi.com/1999-4915/9/12/373/s1. Figure S1: Genome terminal sequences of VINHV and other Dera Ghazi Khan genogroup viruses, Figure S2: Amino acid sequences alignment of glycoprotein Gn of the DGK group viruses and CCHFV, Figure S3: Amino acid sequence alignment of glycoprotein Gc of the DGK group viruses and CCHFV, Figure S4: Amino acid sequence alignment of the nucleoproteins of DGK group viruses and CCHFV, Table S1: Viruses and GenBank accession numbers of sequences used in phylogenetic and other comparative analyses.Table S2: Amino acid sequence identities (%) of nairovirus N proteins as determined by p-distance estimation in MEGA7.
