# Supplementary material for: Genomic Characterisation of Vinegar Hill Virus, An Australian Nairovirus Isolated in 1983 from Argas Robertsi Ticks Collected from Cattle Egrets
Source: Viruses. 2017 Dec 5;9(12):373. doi: 10.3390/v9120373 (PMC5744148; doi:10.3390/v9120373)
Supplement: Supplementary file 1 [file viruses-09-00373-s001.zip › VINHV Supplementary files/Figure_S1.pptx]

## Slide 1
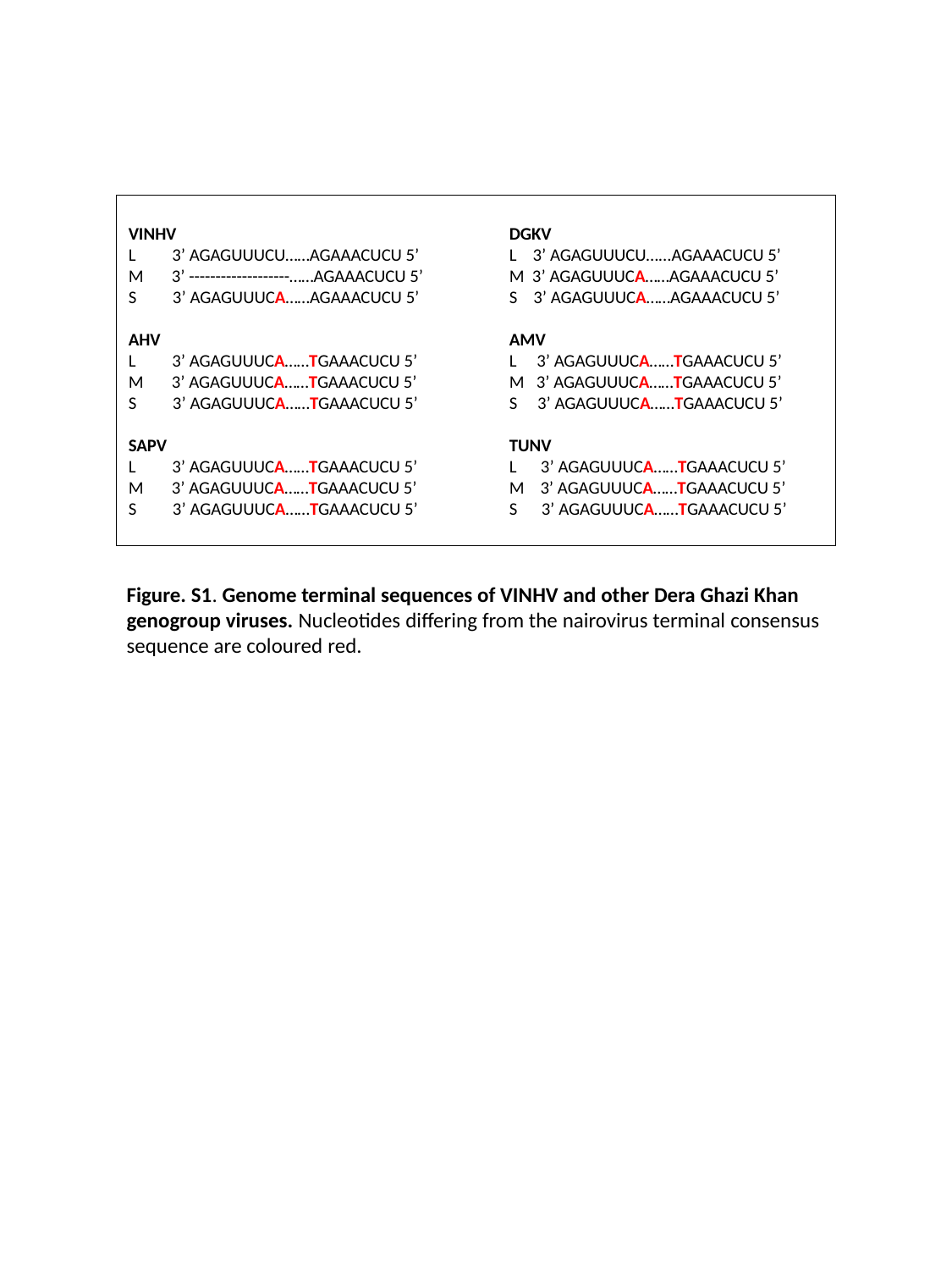

VINHV			DGKV
L 3’ AGAGUUUCU……AGAAACUCU 5’	L 3’ AGAGUUUCU…...AGAAACUCU 5’
M 3’ -------------------……AGAAACUCU 5’	M 3’ AGAGUUUCA……AGAAACUCU 5’
S 3’ AGAGUUUCA……AGAAACUCU 5’	S 3’ AGAGUUUCA……AGAAACUCU 5’
AHV			AMV
L 3’ AGAGUUUCA……TGAAACUCU 5’	L 3’ AGAGUUUCA……TGAAACUCU 5’
M 3’ AGAGUUUCA……TGAAACUCU 5’	M 3’ AGAGUUUCA……TGAAACUCU 5’
S 3’ AGAGUUUCA……TGAAACUCU 5’	S 3’ AGAGUUUCA……TGAAACUCU 5’
SAPV			TUNV
L 3’ AGAGUUUCA……TGAAACUCU 5’	L 3’ AGAGUUUCA……TGAAACUCU 5’
M 3’ AGAGUUUCA……TGAAACUCU 5’	M 3’ AGAGUUUCA……TGAAACUCU 5’
S 3’ AGAGUUUCA……TGAAACUCU 5’	S 3’ AGAGUUUCA……TGAAACUCU 5’
Figure. S1. Genome terminal sequences of VINHV and other Dera Ghazi Khan genogroup viruses. Nucleotides differing from the nairovirus terminal consensus sequence are coloured red.
