# Supplementary material for: Genomic Characterisation of Vinegar Hill Virus, An Australian Nairovirus Isolated in 1983 from Argas Robertsi Ticks Collected from Cattle Egrets
Source: Viruses. 2017 Dec 5;9(12):373. doi: 10.3390/v9120373 (PMC5744148; doi:10.3390/v9120373)
Supplement: Supplementary file 1 [file viruses-09-00373-s001.zip › VINHV Supplementary files/Figure_S2.pptx]

## Slide 1
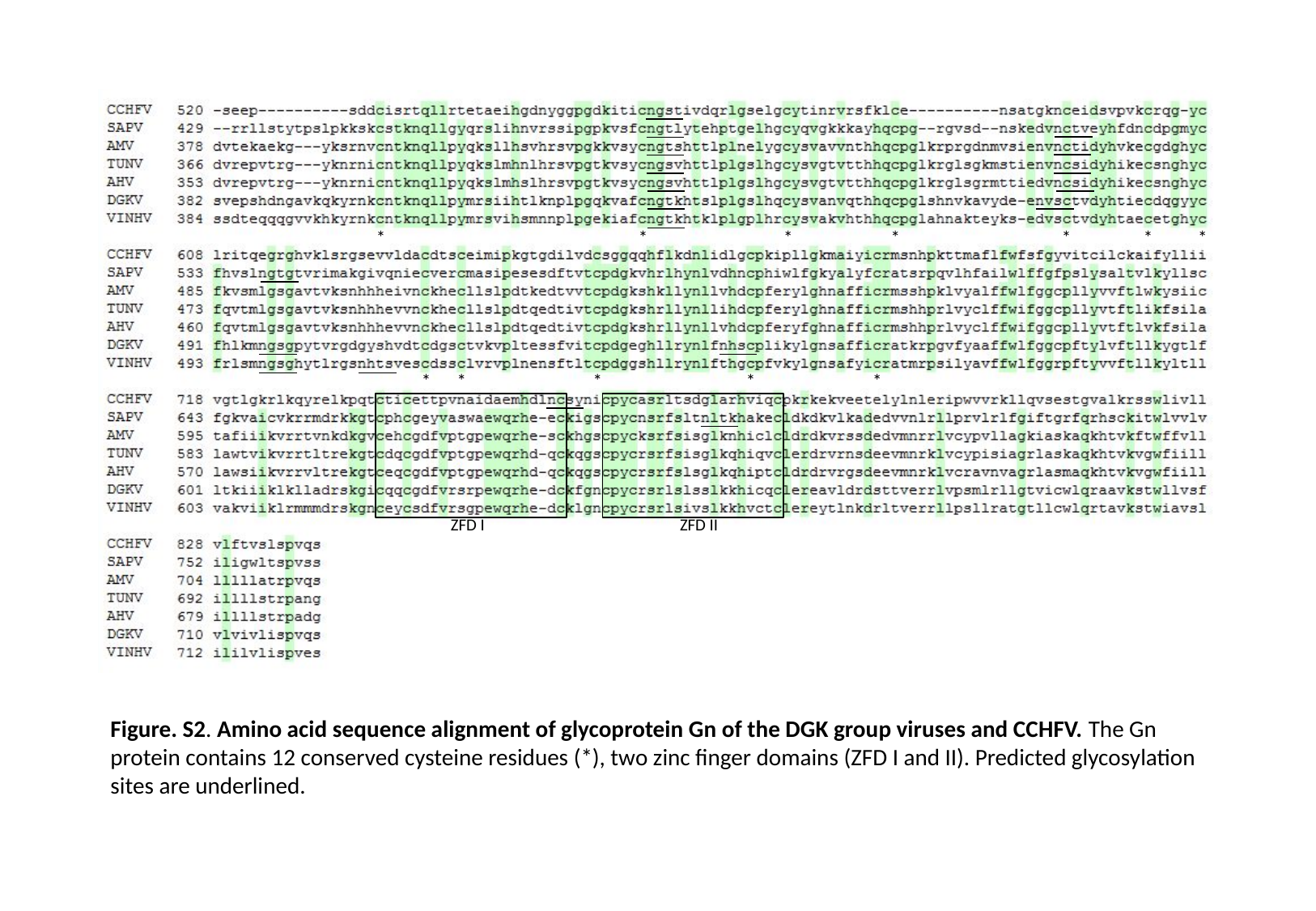

*
*
*
*
*
*
*
*
*
*
*
*
ZFD II
ZFD I
Figure. S2. Amino acid sequence alignment of glycoprotein Gn of the DGK group viruses and CCHFV. The Gn protein contains 12 conserved cysteine residues (*), two zinc finger domains (ZFD I and II). Predicted glycosylation sites are underlined.
