# Supplementary material for: Genomic Characterisation of Vinegar Hill Virus, An Australian Nairovirus Isolated in 1983 from Argas Robertsi Ticks Collected from Cattle Egrets
Source: Viruses. 2017 Dec 5;9(12):373. doi: 10.3390/v9120373 (PMC5744148; doi:10.3390/v9120373)
Supplement: Supplementary file 1 [file viruses-09-00373-s001.zip › VINHV Supplementary files/Figure_S3.pptx]

## Slide 1
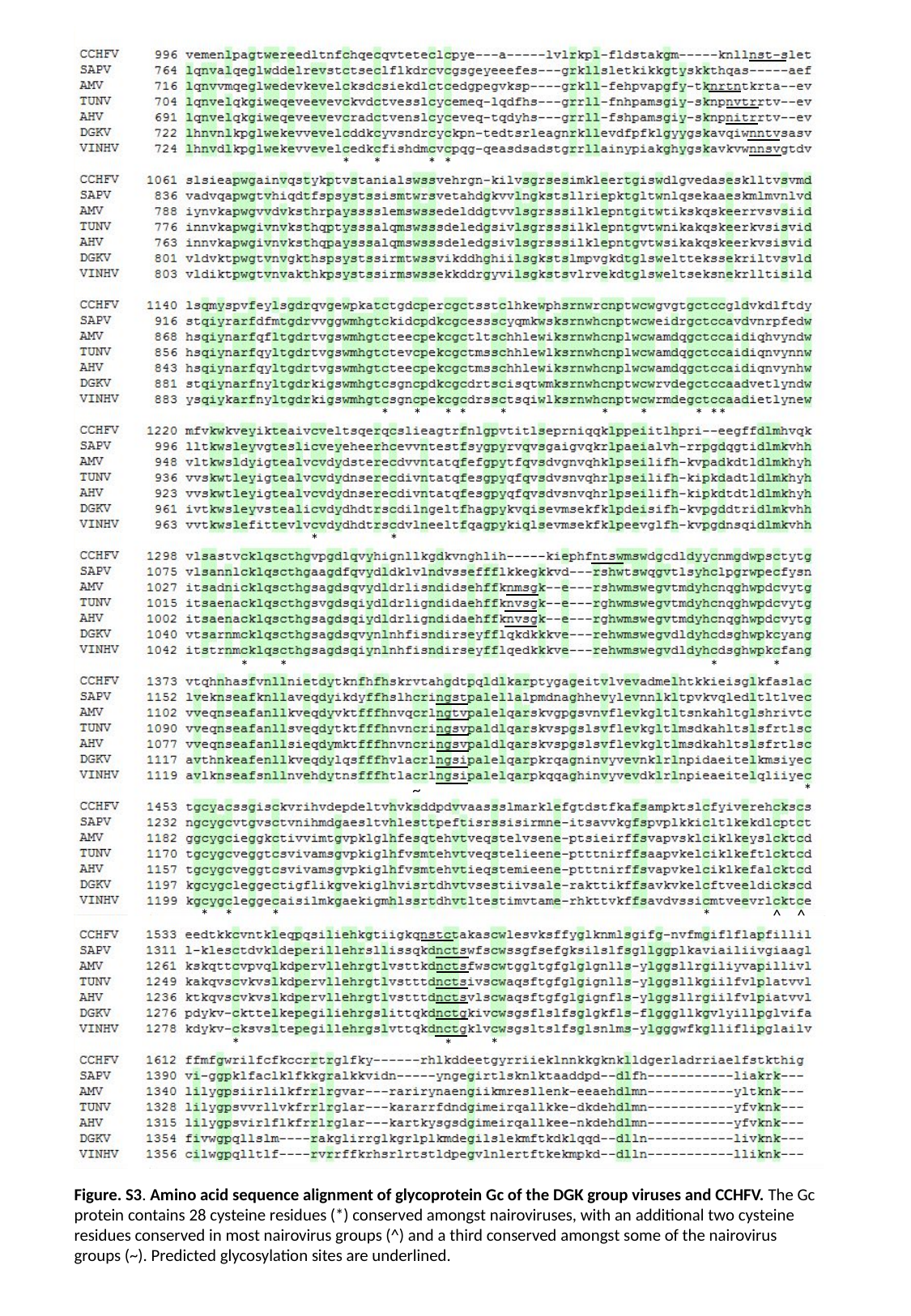

*
*
*
*
*
*
*
*
*
*
*
*
*
*
*
*
*
*
*
*
~
*
*
*
*
*
^
^
*
*
*
Figure. S3. Amino acid sequence alignment of glycoprotein Gc of the DGK group viruses and CCHFV. The Gc protein contains 28 cysteine residues (*) conserved amongst nairoviruses, with an additional two cysteine residues conserved in most nairovirus groups (^) and a third conserved amongst some of the nairovirus groups (~). Predicted glycosylation sites are underlined.
