# Supplementary material for: Genomic Characterisation of Vinegar Hill Virus, An Australian Nairovirus Isolated in 1983 from Argas Robertsi Ticks Collected from Cattle Egrets
Source: Viruses. 2017 Dec 5;9(12):373. doi: 10.3390/v9120373 (PMC5744148; doi:10.3390/v9120373)
Supplement: Supplementary file 1 [file viruses-09-00373-s001.zip › VINHV Supplementary files/Figure_S4.pptx]

## Slide 1
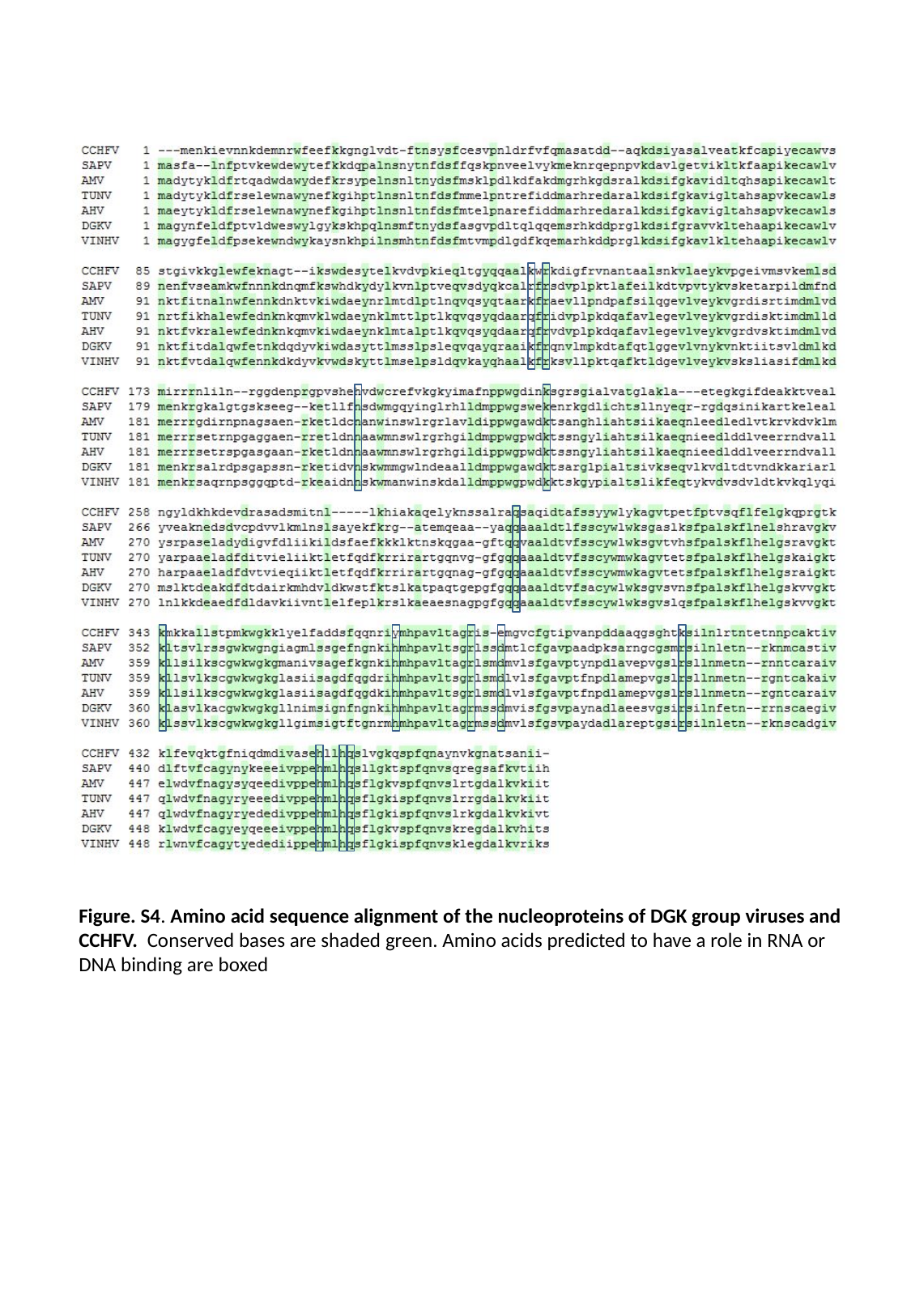

Figure. S4. Amino acid sequence alignment of the nucleoproteins of DGK group viruses and CCHFV. Conserved bases are shaded green. Amino acids predicted to have a role in RNA or DNA binding are boxed
