# Supplementary material for: Genomic Characterisation of Vinegar Hill Virus, An Australian Nairovirus Isolated in 1983 from Argas Robertsi Ticks Collected from Cattle Egrets
Source: Viruses. 2017 Dec 5;9(12):373. doi: 10.3390/v9120373 (PMC5744148; doi:10.3390/v9120373)
Supplement: Supplementary file 1 [file viruses-09-00373-s001.zip › VINHV Supplementary files/Table_S1.pdf]

S1 Table: Viruses and GenBank accession numbers of sequences used in phylogenetic and other comparative analyses

		GenBank accession number		
**Abbreviation**	**Name**	**L seq**	**M seq**	**S seq**
AHV	Abu Hammad virus	KU925434	KU925435	KU925436
AMV	Abu Mina virus	KU925437	KU925438	KU925439
AVAV	Avalon virus	KU925440	KU9255441	KU925442
BDAV	Bandia virus	KU925446	KU9255447	KU925448
CMV	Clo Mor virus	KU343139	KU343140	KU343141
CCHFV	Crimean Congo haemorrhagic virus	NC_005301	NC_005300	NC_005302
DGKV	Dera Ghazi Khan virus	KU925452	KU925453	KU925454
DUGV	Dugbe virus	NC_004159	NC_004158	NC_004157
ERVV	Erve virus	JF911697	JF911698	JF911699
FARV	Farallon virus	KU925461	KU925462	KU925463
GANV	Ganjam virus	KU925464	KU925465	KU925466
GOSV	Gossas virus	KR534878	KR534877	KR534876
GRSV	Great Saltee virus	KU925467	KU925468	KU925469
HAZV	Hazara virus	KP406723	KP406724	KP406725
HTV1	Huangpi tick virus I	KM817667	KM817706	KM817734
HUGV	Hughes virus	KU925470	KU925471	KU925472
IKV	Issyk-Kul virus	KR537441	KR537442	KR537443
KKOV	Kasokero virus	KR537444	KR537445	KR537446
KTRV	Keterrah virus	KR537447	KR537448	KR537449
KUPV	Kupe virus	EU257628	EU257627	EU257626
LPHV	Leopard's Hill virus	NC_025831	NC_025832	NC_025833
NSDV	Nairobi sheep disease virus	EU697949	EU697950	AF504294
PSV	Punta salinas virus	KU925473	KU925474	KU925475
QYBV	Qalyub virus	KU925476	KU925477	KU925478
RAZAV	Raza virus	KU925479	KU925480	KU925481
SAKV	Sakhalin virus	KU925482	KU925483	KU925484
SAPV	Sapphire II	KU925485	KU925486	KU925487
SOLV	Soldado virus	KU925488	KU925489	KU925490
TTV1	Tacheng tick virus I	KM817683	KM817717	KM817743
TAGV	Taggert virus	KU925491	KU925492	KU925493
TFAV	Thiafora virus	KR537450	KR537451	KR537452
TILLV	Tillamook virus	KU925494	KU925495	KU925496
TFLV	Tofla virus	NC_029124	NC_029123	NC_029122
TUNV	Tunis virus	KU925497	KU925498	KU925499
WTV	Wenzhou Tick virus	KM817685	KM817718	KM817745
YOGV	Yogue virus	KR537453	KR537454	KR537455
ZIRV	Zirqa virus	KU925500	KU925501	KU925502
SSV1	Shayang spider virus	NC_031220	NC_031218	NC_031219
