# Supplementary material for: Genomic Characterisation of Vinegar Hill Virus, An Australian Nairovirus Isolated in 1983 from Argas Robertsi Ticks Collected from Cattle Egrets
Source: Viruses. 2017 Dec 5;9(12):373. doi: 10.3390/v9120373 (PMC5744148; doi:10.3390/v9120373)
Supplement: Supplementary file 1 [file viruses-09-00373-s001.zip › VINHV Supplementary files/Table_S2.pdf]

S2 Table. Amino acid sequence identities (%) of nairovirus N proteins determined by p-distance estimation in MEGA7.

	VINHV	DGKV	AHV	TUNV	AMV	SAPV	CCHFV	GANV	NSDV	KUPV	DUGV	HAZV	TFLV	FARV	HUGV	RAZAV	PSV	ZIRV	GRSV	SOLV	WTV	TTV1	HTV1	GOSV	IKV	KTRV	KKOV	LPHV	YOGV	QYBV	BDAV	AVAV	CMV	TILLV	SAKV	TAGV	TFAV
DGKV	72.0																																				
AHV	62.1	59.3																																			
TUNV	62.1	59.9	95.4																																		
AMV	60.6	63.4	76.0	75.8																																	
SAPV	54.4	52.9	52.4	52.4	52.4																																
CCHFV	35.2	35.0	35.9	36.3	37.0	35.5																															
GANV	36.6	35.5	34.8	35.7	35.5	31.7	61.9																														
NSDV	36.6	35.5	34.8	35.7	35.5	31.7	61.9	100.0																													
KUPV	35.2	34.6	34.1	33.7	36.3	32.4	58.1	63.2	63.2																												
DUGV	35.0	33.9	32.6	33.5	33.7	31.5	56.8	61.0	61.0	75.6																											
HAZV	36.6	34.4	37.9	37.7	35.7	34.8	60.8	63.2	63.2	57.9	56.2																										
TFLV	35.7	33.7	36.1	35.9	35.0	32.8	59.3	62.1	62.1	55.5	54.8	76.0																									
FARV	42.1	43.6	44.3	44.3	43.0	41.0	31.5	31.1	31.1	31.3	30.6	32.2	30.8																								
HUGV	41.9	43.4	44.3	44.3	43.2	41.0	31.3	31.5	31.5	31.5	30.8	32.2	30.8	99.1																							
RAZAV	41.4	42.7	43.2	43.2	43.4	41.0	32.6	32.6	32.6	32.6	32.2	33.0	32.4	82.2	81.5																						
PSV	41.4	42.7	44.1	43.6	42.7	39.2	32.2	31.7	31.7	32.8	31.5	33.3	31.7	85.7	85.0	83.0																					
ZIRV	41.6	44.5	43.6	43.6	43.8	40.3	32.2	31.7	31.7	32.8	31.9	32.8	31.1	76.7	76.7	72.7	73.6																				
GRSV	41.2	42.1	42.1	41.6	41.9	40.7	31.1	31.1	31.1	33.0	31.9	30.2	31.1	59.3	59.3	58.1	57.0	60.6																			
SOLV	42.1	40.3	42.7	42.7	41.2	41.2	31.3	32.4	32.4	32.8	32.8	32.2	31.3	60.8	61.0	58.8	59.0	59.7	80.6																		
WTV	32.6	30.6	32.8	32.4	31.5	31.5	34.8	33.9	33.9	31.3	33.5	34.8	34.4	31.7	31.5	31.9	31.9	31.5	31.9	31.5																	
TTV1	32.4	32.2	33.9	33.5	34.1	32.8	34.6	36.6	36.6	34.4	32.8	36.3	34.8	30.8	31.1	32.2	31.9	31.1	33.0	31.5	50.9																
HTV1	33.5	32.8	31.9	31.5	33.0	33.5	35.2	34.8	34.8	35.0	34.4	34.1	32.2	30.2	30.0	29.5	29.7	30.8	31.3	30.6	48.2	52.2															
GOSV	34.4	36.6	35.5	34.8	35.5	35.2	34.6	34.8	34.8	33.0	32.6	33.9	31.9	33.0	32.8	32.4	33.7	32.6	28.0	30.0	30.6	34.1	31.9														
IKV	34.1	34.1	35.5	34.1	35.2	36.3	35.2	35.7	35.7	35.2	33.5	34.6	33.7	32.8	33.0	33.7	33.3	33.3	30.4	31.9	31.1	33.3	29.3	63.7													
KTRV	33.5	34.8	35.2	34.6	35.7	35.2	36.3	35.0	35.0	33.3	32.4	34.1	34.1	32.6	32.6	33.0	33.7	33.0	29.3	30.8	30.8	32.8	30.2	66.7	86.8												
KKOV	38.1	38.3	37.2	35.7	37.4	38.8	37.7	37.4	37.4	37.0	38.1	36.1	36.6	32.2	32.2	31.7	31.5	32.2	32.4	33.0	33.7	34.6	35.0	46.5	47.1	47.8											
LPHV	36.8	37.7	37.2	35.7	36.3	38.8	38.1	37.4	37.4	37.7	37.9	35.9	36.8	32.4	32.4	32.2	31.9	31.9	32.2	32.6	33.7	34.8	35.0	47.6	46.5	47.4	91.6										
YOGV	38.3	39.9	38.1	37.0	38.5	38.8	37.0	37.4	37.4	37.7	36.8	34.4	34.4	32.8	33.0	31.9	32.4	32.2	32.8	33.0	35.0	34.8	35.2	47.8	48.0	47.6	80.6	80.0									
QYBV	35.2	36.1	33.5	32.6	35.5	36.1	37.2	38.3	38.3	35.7	36.8	36.1	36.1	30.4	30.6	31.3	30.4	30.4	29.7	29.7	35.0	33.7	33.7	41.9	42.1	40.3	50.9	50.2	49.8								
BDAV	35.5	36.1	32.8	32.2	35.7	37.7	37.7	36.1	36.1	34.6	36.3	34.1	35.9	30.2	30.2	32.4	31.5	30.6	29.3	29.7	34.6	34.4	32.6	43.6	42.1	42.1	50.9	51.5	51.8	72.9							
AVAV	35.2	34.4	37.7	36.6	36.1	35.2	43.2	41.0	41.0	41.4	43.0	44.3	42.1	32.6	32.4	33.9	33.0	34.6	31.7	31.7	35.5	37.4	37.4	37.9	38.3	39.0	42.7	43.2	39.4	37.7	37.7						
CMV	33.5	33.0	33.5	33.7	33.3	33.7	43.0	42.5	42.5	41.4	41.6	42.5	43.8	30.6	30.6	32.4	31.7	31.3	31.1	31.7	34.1	35.5	38.1	37.7	38.1	37.0	38.1	39.0	36.6	38.1	37.4	64.8					
TILLV	31.9	32.6	32.8	32.2	33.5	32.8	42.3	42.5	42.5	41.2	41.4	43.0	43.4	30.4	30.6	33.0	31.7	32.2	30.4	30.6	34.1	35.2	37.4	35.9	37.9	36.6	37.7	39.2	36.1	37.2	36.3	64.3	84.1				
SAKV	31.7	32.6	32.6	31.9	33.3	32.4	42.3	42.1	42.1	41.0	41.2	43.0	43.4	30.8	31.1	33.0	32.2	32.6	30.2	30.8	33.7	34.8	37.7	35.9	37.4	36.1	37.4	39.0	35.9	36.8	36.3	63.7	84.1	98.9			
TAGV	32.8	32.6	34.4	33.3	32.6	33.5	43.8	43.0	43.0	42.3	42.3	43.2	43.2	30.4	30.4	33.3	31.7	31.9	30.4	30.0	35.9	35.7	38.5	35.5	37.4	36.1	35.7	36.8	34.6	36.3	37.2	64.8	78.4	80.0	79.3		
TFAV	32.2	30.2	33.3	33.3	33.3	32.8	44.5	44.7	44.7	43.6	44.1	42.3	44.3	31.1	31.1	32.2	31.7	32.4	31.3	33.0	30.0	32.6	34.4	32.6	33.0	32.6	37.0	37.4	37.2	33.0	34.4	38.5	39.4	38.8	38.5	39.2	
ERVV	32.4	31.3	32.6	31.5	31.7	29.5	44.1	43.0	43.0	42.3	44.1	41.6	44.3	31.1	31.1	32.8	31.7	32.6	30.4	32.6	30.6	31.9	33.7	31.9	34.8	33.5	32.6	33.3	32.2	30.8	30.8	37.7	38.3	38.1	37.9	40.3	70.3
